# Specific Wheat Fractions Influence Hepatic Fat Metabolism in Diet-Induced Obese Mice

**DOI:** 10.3390/nu11102348

**Published:** 2019-10-02

**Authors:** Daniela Graf, Karolin Weitkunat, Andreas Dötsch, Gerhard Liebisch, Maik Döring, Ralf Krüger, Dominic Stoll, Elisabeth Vatareck, Elena von Coburg, Gunnar Loh, Bernhard Watzl

**Affiliations:** 1 Department of Physiology and Biochemistry of Nutrition, Max Rubner-Institut, 76131 Karlsruhe, Germany; 2 Department of Physiology of Energy Metabolism, German Institute of Human Nutrition Potsdam-Rehbruecke, 14558 Nuthetal, Germany; 3 Institute of Functional Interfaces, Karlsruhe Institute of Technology, 76344 Eggenstein-Leopoldshafen, Germany; 4 Institute of Clinical Chemistry, University Hospital Regensburg, 93053 Regensburg, Germany; 5 Department of Safety and Quality of Fruit and Vegetables, Max Rubner-Institut, 76131 Karlsruhe, Germany

**Keywords:** wheat, whole grain, bran, aleurone, microbiota, SCFA, lipid metabolism, obesity

## Abstract

Low whole grain consumption is a risk factor for the development of non-communicable diseases such as type 2 diabetes. Dietary fiber and phytochemicals are bioactive grain compounds, which could be involved in mediating these beneficial effects. These compounds are not equally distributed in the wheat grain, but are enriched in the bran and aleurone fractions. As little is known on physiological effects of different wheat fractions, the aim of this study was to investigate this aspect in an obesity model. For twelve weeks, C57BL/6J mice were fed high-fat diets (HFD), supplemented with one of four wheat fractions: whole grain flour, refined white flour, bran, or aleurone. The different diets did not affect body weight, however bran and aleurone decreased liver triglyceride content, and increased hepatic n-3 polyunsaturated fatty acid (PUFA) concentrations. Furthermore, lipidomics analysis revealed increased PUFA concentration in the lipid classes of phosphatidylcholine (PC), PC-ether, and phosphatidylinositol in the plasma of mice fed whole grain, bran, and aleurone supplemented diets, compared to refined white flour. Furthermore, bran, aleurone, and whole grain supplemented diets increased microbial α-diversity, but only bran and aleurone increased the cecal concentrations of short-chain fatty acids. The effects on hepatic lipid metabolism might thus at least partially be mediated by microbiota-dependent mechanisms.

## 1. Introduction

During recent decades, obesity and its associated diseases have spread world-wide. According to the WHO, 39% of adults were overweight and 13% were obese in 2016 [1]. Amongst the diseases associated with obesity are cardiovascular diseases, stroke, diabetes, some cancers, and non-alcoholic fatty liver disease (NAFLD) [2,3], of which ischemic heart disease and stroke are the leading causes of death world-wide [4].

Nutrition is generally seen as one important factor in the prevention of obesity and obesity-associated diseases. According to the latest Global Burden of Disease Study, 22% of all deaths were attributed to unhealthy diets [5]. Low whole grain consumption has been identified as the leading dietary risk factor contributing to the global burden of disease in various countries all over the world, including Brazil, Germany, India, and the USA [5]. As cereal grains provide approximately 30% of total energy intake [6], they have a huge impact on diet quality.

Whole grain has been specifically associated with beneficial health effects. Several meta-analyses could show that an increased whole grain intake is associated with a decreased risk for the incidence and/or mortality of type 2 diabetes, cardiovascular diseases, and cancer, as well as a reduced risk for all-cause mortality [7,8,9,10,11].

Constituents that play a role in the health promoting effects of whole grain are dietary fiber, phytochemicals, minerals, and vitamins [12]. The role of dietary fiber and phytochemicals has been the focus of recent research. The gut microbiota ferments dietary fiber to short-chain fatty acids (SCFAs), which serve as energy substrates for intestinal epithelial cells, which are metabolized in the liver, and affect physiological processes throughout the body [13]. Amongst the effects discussed to be mediated by SCFA are increased insulin sensitivity and reduced hepatic fat storage [14].

Recently, betainized compounds have been identified as microbial metabolites following whole grain consumption, and these compounds are being discussed as further potential mediators of microbial effects [15].

Furthermore, dietary fiber can influence microbiota composition. As reviewed recently, the intake of intact cereal fiber can improve bacterial diversity, which is associated with better health [16].

Diets with a high-fiber content have also been recommended to people suffering from obesity-associated pathologies, such as insulin resistance or liver diseases, as they can improve glycemic response, and reduce cholesterol [17].

As reviewed recently, whole grains contain a plethora of phytochemicals, which have been associated with various health-promoting effects, such as anti-cancer, anti-diabetes, and cardioprotective potentials [18]. Phytochemicals further interfere with the intestinal microbiota in multiple ways [19].

The term “whole-grain products” is not defined consistently. However, the general understanding is that whole grain products contain all fractions of the grain in the same proportions as the intact kernel, with the main fractions being the starchy endosperm, the bran, and the germ [12]. The distribution of the bioactive constituents in the different grain fractions varies [12], explaining the higher-nutritional value of whole grains compared to refined grains. For wheat, the largest fraction of the kernel makes up the endosperm (80–85%), which contains mostly starch, some proteins and fiber [20]. The bran fraction makes up 13–19% of the kernel, and contains most of the dietary fiber (approximately 50% of the bran consists of dietary fiber) [21]. It can be further divided according to the layers it consists of. Aleurone, a unicellular layer, which is the innermost layer adjacent to the endosperm, constitutes the largest bran fraction (50%) with approximately 50% dietary fiber, and 10% mineral content [20,22]. As dietary fiber plays a role in mediating the health-promoting effects of whole grain, the defined biological activities of its subfraction aleurone, as well as of wheat bran, have received special interest.

Although wheat is one of the most important staple crops world-wide [23], studies investigating the effects of different wheat fractions are missing, even though it is well known that the concentrations of bioactive compounds differ in these fractions. Therefore, the aim of this study was to investigate, which wheat fraction(s) mediate the physiological effects of whole grain wheat.

## 2. Materials and Methods

### 2.1. Study Design and Diets

Male C57BL/6J mice (*n* = 40) were purchased from Charles River (Sulzfeld, Germany) at the age of eight weeks. The mice were single-caged in individually ventilated cages (cage type: 1284; Tecniplast IVC Blue Line Classic). After one week of acclimation they were randomly allocated into 4 groups (*n* = 10/group). Each group received one of four high-fat diets (HFD) (ssniff, Germany), with 19% w:w fat content, and 10% w:w of a wheat grain fraction, which was either whole grain flour (WGD), white flour (WFD), bran (BRD), or aleurone (ALD) (Table 1). The grain flours and bran were kindly provided by Dr. Jens Begemann, Max Rubner-Institut, and the aleurone was kindly provided by the Bühler AG, Switzerland. The mice were fed their respective diets and water for 12 weeks *ad libitum*. Diet intake and body weight was monitored throughout the study. After the 12 weeks the mice were euthanized. Blood was collected via cardiac puncture from the anesthetized mice. After the death of the mice was confirmed, liver, adipose tissue, and cecum content were collected, weighed, and snap frozen. All tissues were stored at −80 °C until further analysis. The experiments were divided in two time periods: Phase 1 (with 6/group) and phase 2 (with 4/group).

### 2.2. Gene Expression

Isolation of RNA, cDNA synthesis, and qRT-PCR were performed as previously described [24]. Gene expression was calculated as ΔΔCt, using the 18S rRNA gene as a reference. Data were expressed relative to the WGD group and normalized to a value of 1. The primer and probe sequences are summarized in Table S1.

### 2.3. Quantification of Plasma Lipid Species

Lipids were quantified by direct flow-injection electrospray ionization tandem mass spectrometry (ESI-MS/MS) in positive ion mode, using the analytical setup and strategy described previously [25,26]. Lipid extraction was performed according to the method of Bligh and Dyer [27], in the presence of not naturally occurring lipid species as internal standards. The following lipid species were added as internal standards: PC 14:0/14:0, PC 22:0/22:0, PE 14:0/14:0, PE 20:0/20:0 (di-phytanoyl), PI 17:0/17:0, LPC 13:0, LPC 19:0, Cer d18:1/14:0, Cer 17:0, D7-FC, CE 17:0, and CE 22:0.

A fragment ion of m/z 184 was used for phosphatidylcholine (PC), sphingomyelin (SM) [26], and lysophosphatidylcholine (LPC) [28]. Neutral loss fragments were used for the following lipid classes: Phosphatidylethanolamine (PE), and phosphatidylinositol (PI), with a loss of 141 and 277, respectively [29,30]. PE-based plasmalogens (PEP) were analyzed according to the principles described by Zemski–Berry [31]. Sphingosine based ceramides (Cer) and hexosylceramides (HexCer) were analyzed using a fragment ion of m/z 264 [32]. Free cholesterol (FC) and cholesteryl ester (CE) were quantified using a fragment ion of m/z 369, after selective derivatization of FC [25]. Quantification was achieved using two non-naturally occurring internal standards (IS) for each lipid class (except for PI, SM was calculated using PC IS, and PE-based plasmalogens were calculated using PE IS) and calibration lines generated by the standard addition of a number of naturally occurring species to the plasma. Calibration lines were generated for the following naturally occurring species: PC 34:1, 36:2, 38:4, 40:0, and PC O-16:0/20:4, SM d18:1/16:0, 18:1, 18:0; LPC 16:0, 18:1, 18:0; PE 34:1, 36:2, 38:4, 40:6, and PE P-16:0/20:4, Cer d18:1/16:0, 18:0, 20:0, 24:1, 24:0, FC, CE 16:0, 18:2, 18:1, 18:0. Deisotoping and data analysis for all lipid classes were performed by self-programmed Excel macros, as described previously [26,33]. Lipid species were annotated according to the proposal for shorthand notation of lipid structures that are derived from mass spectrometry [34]. Glycerophospholipid species annotation is based on the assumption of even-numbered carbon chains only. SM species annotation is based on the assumption that a sphingoid base with two hydroxyl groups is present.

### 2.4. Analysis of Liver Lipids

#### 2.4.1. Liver Triglyceride Analysis

Hepatic triglyceride concentration was measured in 40 mg of ground liver tissue, after extraction with a 10 mmol/L sodium phosphate buffer (pH 7.4), containing 1 mmol/L EDTA and 1% polyoxyethylene (10) tridecyl ether, according to manufacturer instructions (Triglyceride Determination Kit, Sigma-Aldrich). Triglyceride concentrations were normalized to protein content (DC Protein Assay; Bio-Rad Laboratories GmbH, München, Germany).

#### 2.4.2. Long-Chain Fatty Acid Analysis

Analyses of the FA spectra of liver phospholipids were performed with a modified method, using extraction with tert-butyl methyl ether/methanol (2/1, *v*/*v*), solid-phase extraction (SPE columns containing 100 mg aminopropyl-modified silica; Chromabond, MachereyNagel GmbH & Co. KG, Germany), hydrolysis and methylation with trimethyl sulfonium hydroxide (TMSH), and subsequent analysis by GC (Agilent GC system 7890A, equipped with Agilent 7000 GC/MS Triple Quad, and a flame ionization detector (FID)) as described previously [35].

### 2.5. Microbiota

#### 2.5.1. DNA Extraction

For the analysis of the intestinal microbiota, DNA was isolated from caecum contents. Samples were placed in 2 mL tubes of Lysing Matrix A (MP Biomedicals), mixed with a 350 µL lysis buffer, and then homogenized using the FastPrep instrument (MP biomedicals) for 40 seconds on power level 6. The suspension was centrifuged for 10 min at 16,000 rcf, with the supernatant transferred to a fresh tube and centrifuged again for 5 min at 16,000 rcf. The resulting supernatant was further treated with the QIAamp DNA mini kit (Qiagen, Hilden, Germany) according to the manufacturer’s instructions, with the modification of repeating the washing step using buffer AW2, and eluting twice in 50 µL nuclease-free water. The final DNA concentrations were determined using the Qubit fluorometer with the dsDNA HS and dsDNA BR kits (Thermo Fisher Scientific, Inc., Waltham, MA, USA).

#### 2.5.2. 16S Amplicon Sequencing

Amplicons of the V1-V2 region of the bacterial 16S rRNA gene were prepared using the primer design described by Camarinha–Silva et al. [36], which achieves multiplexing by employing a combination of custom 6-mer barcode sequences directly adjacent to the forward 16S sequence, and the adapter-integrated index sequences commonly used by Illumina multiplexing kits. See supplementary Table S2 for a complete list of primer sequences used for amplicon sequencing in this study. Amplification and integration of the multiplexed adapters were performed in three PCR steps: (1) Enrichment PCR: Initially the target region of the 16S rRNA gene was amplified with primers 27F and 338R described elsewhere [37,38] in a total volume of 20 µL containing 0.2 µM of each primer, 0.2 mM dNTPs, 0.5 U of PrimeSTAR HS DNA Polymerase (Takara), and 2 µL DNA template using a PCR program with a 3 min initial denaturation at 95 °C, with 10 cycles of denaturation at 98 °C (10 seconds), annealing at 55 °C (10 seconds), elongation at 72 °C (45 seconds), and final elongation at 72 °C for 5 min. (2) Custom barcode integration PCR: 1 µL of the product of the enrichment PCR was used as a template for the 2nd PCR with barcoded primers IlluFBCxx and IlluRevAdap, and identical concentrations and program, as in the enrichment PCR. (3) Illumina multiplexing PCR: In the final PCR, 1 µL of the product of the barcode integration PCR was used as template for the 3rd PCR with an Illumina Multiplexing primer and Index-xx primer, and with the identical concentrations and program as in the previous steps. Final PCR products were purified with magnetic HighPrep beads (MagBio Genomics) and precipitated (to increase the concentration) by adding 2.5 volumes of ethanol, 0.1 volumes of sodium acetate (3 M, pH 5.2), and incubation over night at −20 °C. The precipitate was centrifuged at 16,060 rcf for 10 min and the pellet resuspended in 20 µL Tris-HCl (pH 8.0) at 37 °C, with shaking for 35 min.

Samples were merged at equal molarity to 4 sequencing libraries, each of which was sequenced on an Illumina MiSeq instrument using v3 chemistry with 2 × 300 cycles, following the manufacturer’s instructions, and supplemented with 20% of PhiX DNA.

#### 2.5.3. Bioinformatic Analysis of Microbiome Data

Sequence raw data in FASTQ format were demultiplexed using a custom Perl script according to their 6 nt custom barcode sequence, thereby removing 16S primer sequences and barcodes. Afterwards, the remaining reads were processed using *mothur* version 1.41.1 [39], following the MiSeq SOP [40]. Briefly, paired reads were joined to contigs, filtered to remove contigs with ambiguous base calls and homopolymers longer than 12 nt, and aligned with the Silva database SSU Ref NR 99 v132 [41]. The sequences were pre-clustered allowing for 3 mismatches (i.e., ~1 per 100 nt), and chimeric sequences were removed using the VSEARCH algorithm [42]. Finally, the sequences were classified using the Silva v132 taxonomy, with the sequences classified as eukaryote, mitochondria, chloroplasts, or unclassified at the domain level removed, and with operational taxonomic units (OTUs) picked at a 97% identity level.

The composition of the microbiota was further analyzed using the R software (version 3.6.1) with the packages: *Phyloseq* [43], *vegan* [44], *pairwiseAdonis* [45], and *ggplot2* [46]. The alpha-diversity of samples was estimated using the Shannon and inverse Simpson indices, and calculated by the *phyloseq* function *estimate_richness*. Abundance data was filtered to reduce noise by removing OTUs that had a maximum abundance of less than 1% in any sample, resulting in a remaining set of 55 OTUs. Principal coordinates analysis (PCoA) was performed using the *ordinate* function, with Jensen–Shannon-divergence (JSD) as the distance metric. The differences in the distribution of samples between the groups were tested using permutational multivariate analysis of variance (PERMANOVA), implemented in the *adonis* and *pairwiseAdonis* functions. Correlations between SCFA concentrations and OTU abundances were calculated using the R function *cor.test*. A phylogenetic tree of the OTUs was calculated based on a multi-sequence alignment of the 55 OTUs using *Clustal W* [47].

#### 2.5.4. Data Availability

The sequence data obtained in this study have been deposited in the NCBI Sequence Read Archive (https://trace.ncbi.nlm.nih.gov/Traces/sra/) as a BioProject with the accession no. PRJNA545049.

### 2.6. SCFA Analysis

SCFA in the cecum was analyzed by GC-FID without derivatization. The mice cecum (4 to 26 mg) was freeze-dried using an Alpha 1-2 freeze-drying manifold from Martin Christ (Osterode, Germany). 20 µL internal standard (ISTD, 4-ethylbutyric acid, 1 g/L) was added before the samples were dissolved in 100 µL HCl (25%) (Merck, Germany), and further diluted with water to an end volume of 500 µL. Samples were placed in an overhead mixer for 10 min, followed by vortexing for 10 min, and then centrifugation for 10 min at 15,000 rpm. The supernatant was transferred to another tube. The extraction was completed by adding 500 µL water, vortexing (10 min), and centrifugation (10 min at 15,000 rpm). The supernatants were combined, mixed, and centrifuged once again (10 min at 15,000 rpm). 200 µL were transferred to conic GC vials. Samples were analyzed using a 6890 GC from Agilent (Waldbronn, Germany), equipped with a split/splitless injector. A 0.5 µL sample was injected using a split ratio of 1:10 at 200 °C. SCFAs were separated on a ZB-Wax column with the dimensions 24 m × 0.25 mm × 0.25 µm (Phenomenex, Aschaffenburg, Germany). Hydrogen was used as carrier gas with a flow rate of 40 cm/sec. The following temperature program was used: 50 °C (start; 0 min)/50 to 230 °C (10° C/min; 18 min)/230 (isocratic; 20 min) Individual SCFAs were detected by FID at 230 °C. Compounds were quantified by internal calibration (6 levels). All SCFA standards were obtained from Sigma-Aldrich (Taufkirchen, Germany). Matrix controls (2 levels) were used to monitor method performance and for batch correction.

### 2.7. Histology

For histological analysis of adipose tissue, frozen visceral adipose tissue (VAT) was formalin fixed, paraffin embedded, sectioned into 5 µm sections, and hematoxylin/eosin stained. The average adipocyte density was determined by measuring the perimeter around 500 adipocytes, from at least 4 different fields, at 200 × magnification. Adipose tissue sections were viewed with an Axiovert S100 microscope (Zeiss, Jena, Germany), equipped with an Axiocam 105 color Digital Camera and the Zen 2 core software version 2.5 (both Zeiss, Jena, Germany). For the measurements ImageJ (NIH, Bethesda, MD, USA) was used.

### 2.8. Statistical Analysis

Data are shown as mean ± standard deviation (SD). Single variables were analyzed with nonparametric methods based on ranks. Van der Waerden scores (transformation of the empirical distribution to standard normal, ignoring any group membership) were used as the input of an Anova model, with diet type and phase as grouping factors. An adjustment of the phase was necessary due to variations between the two time periods. To indicate differences between the diet types an F-test was performed. Further post-hoc Tukey tests were conducted for pairwise comparisons. Model checking for variance homogeneity were based on the Levene test. For a sound discussion about nonparametric analysis of variance methods see for example [48]. The 30 lipids with the smallest p-value of the F-test were chosen to perform a principle component analysis on the centered and to unit variance scaled data. Statistical analyses were performed with the software *R* [49], using the packages *multcomp* [50] and *ggplot2* [46]. Statistical significance was set at the 0.05 level.

## 3. Results

All statements in this section are made under these definitions: All results are presented as means ± SD. Differences are only designated as such, when they are statistically significant (*p* ≤ 0.05).

### 3.1. Impact of Different Wheat Fractions on Food Intake, Body Weight, and Organ Weights

Food intake and body weights were monitored throughout the 12 weeks of the intervention period. The average daily food intake did not differ between the intervention groups (Table 2), and neither did the final body weight after 12 weeks of feeding intervention. The mice weighed approximately 36 g, independent of the diet group (Table 2). As obesity is associated with increased weight of adipose tissue and the hypertrophy of adipocytes [51], we assessed the weight of various adipose tissue depots (epididymal, subcutaneous, visceral) and adipocyte size (measured as adipocyte density) in the visceral adipose tissue. However, the different grain fractions neither influenced adipose tissue weight, nor adipocyte density (Table 2).

**Figure 1:**
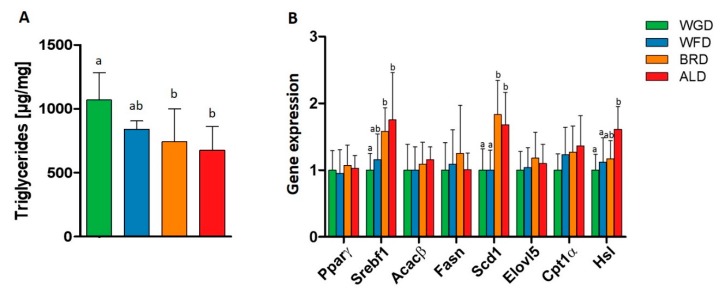
Liver triglyceride content (**A**), and liver gene expression of transcription factors and enzymes involved in liver lipid metabolism (**B**), in C57BL/6J mice fed with WGD, WFD, BRD, or ALD for 12 weeks. ^ab^ marked bars without a common letter differ significantly (*p* < 0,05). Data presented are mean ± SD. *n* = 7–8/group; ACAC-β: Acetyl-CoA Carboxylase β; ALD: Aleurone supplemented diet; BRD: Bran supplemented diet; Cpt1α: Carnitine palmitoyltransferase 1α; Elovl5: Fatty acid elongase 5; Fasn: Fatty acid synthase; Hsl: Hormone-sensitive lipase; Scd1/SCD1: Stearoyl-CoA desaturase 1; SREBF-1: Sterol regulatory element-binding transcription factor 1; WFD: White flour supplemented diet; WGD: Whole grain wheat supplemented diet.

Obesity results in ectopic fat storage in the liver, leading to fatty liver diseases [2]. Even though the liver weight did not differ between the intervention groups (Table 2), supplementation of the diets with bran and aleurone reduced triglyceride concentrations in the liver compared to the whole grain wheat (Figure 1A), thus indicating that bran and aleurone might have a hepatoprotective potential.

### 3.2. Lipid Metabolism

To further investigate the underlying mechanisms, we measured the gene expression of the enzymes and transcription factors involved in lipid metabolism and its regulation (Figure 1B). Both the BRD and ALD increased the gene expression of sterol regulatory element-binding transcription factor 1 (SREBF-1) compared to the WGD, and the expression of stearoyl-coA desaturase (SCD1), compared to the WGD and WFD, but only the ALD increased the gene expression of hormone-sensitive lipase (HSL) compared to the WGD and WFD.

SCD1 catalyzes the introduction of a double-bond into saturated fatty acids (SFAs). Therefore, increased expression of SCD1 might influence the concentrations of SFA, monounsaturated fatty acid (MUFA), and polyunsaturated fatty acids (PUFAs). Hence, we investigated if the composition of fatty acids in liver lipid extracts was influenced by the intervention (Table 3), to see if increased expression of SCD1 led to reduced SFA and increased MUFA concentrations. In contrast to these expectations, concentrations of the long-chain SFA were not influenced by the intervention and the ALD reduced the concentration of the MUFA 18:1n9c (oleic acid) compared to the WGD and WFD (Table 3). Furthermore, the intervention had an impact on the concentrations of several PUFAs. The ALD increased the concentrations of C18:2n6t (trans-linoleic acid) compared to the WFD, and reduced the concentration of C22:5n6 (docosapentaenoic acid) compared to the WGD (Table 3). However, the results for trans-linoleic acid need to be interpreted with caution, due to the very low concentrations. The BRD decreased concentration of both n6 PUFA C22:4n6 and C22:5n6, whereas it increased the concentration of the n3 PUFA 22:6n3 (docosahexaenoic acid (DHA)) compared to the WGD and WFD (Table 3). An increase in DHA was also observed for the ALD compared to the WGD and WFD (Table 3). These changes lead to an increase in total n3 PUFA and a decreased ratio of n6/n3 fatty acids in the liver of mice fed the ALD and BRD, compared to the WGD.

Taken together, the results suggest that supplementation of diets with aleurone and bran can reduce liver triglyceride content and increase n3 PUFA, compared to whole grain and refined white wheat flour; and thus, they might have a hepatoprotective effect. However, no differences were observed between whole grain and white wheat flour.

**Figure 2:**
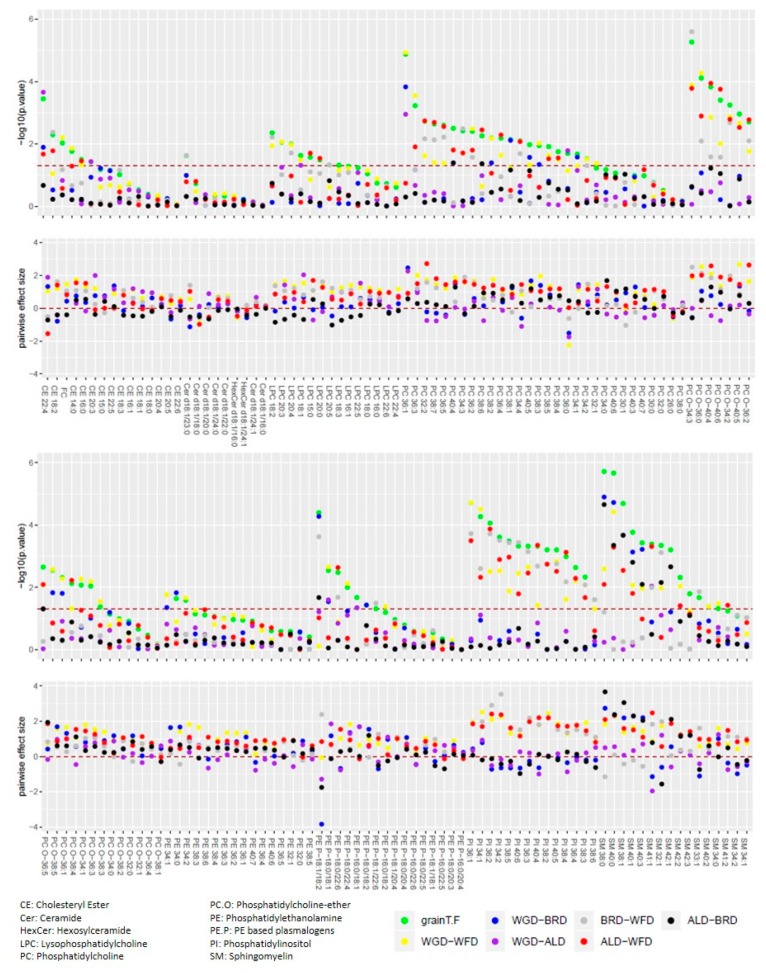
*p*-values and effect sizes for plasma lipids sorted by class. Upper panel: For each lipid the p-values are represented for the F-test (green dots), indicating differences between the diets and for the post-hoc Tukey test, indicating pairwise comparisons (other colored dots). Lower panel: The pairwise Cohen’s effect size (difference between the two means divided by the pooled standard deviation) is given.

We further assessed if the wheat fractions affected lipid metabolism in the liver only, or if systemic effects could be observed as well. Therefore, lipidomics analysis of plasma was conducted. The intervention influenced plasma phospholipid composition. As depicted in Figure 2, differences were mainly observed for phosphatidylcholine (PC) and phosphatidylcholine-ether (PC.O), phosphatidylinositol (PI) and sphingomyelin (SM). For each lipid the p-values are represented for the F-test, indicating differences between the diets and for the post-hoc Tukey test, indicating pairwise comparisons. All PI quantified in this study were affected by the dietary intervention (green dots). Pair-wise comparisons revealed that differences mainly occurred between WFD and the other groups (red dots: WFD‑ALD, yellow dots: WFD‑WGD, grey dots: WFD‑BRD). Similar results were observed for PCO and PC. Approximately two-thirds of the components in these lipid classes were affected by the intervention, and again the WFD differed from the other groups for many of the quantified components. Interestingly for SM, half of the components affected by the diet showed differences between the ALD and the BRD, even though hardly any differences could be observed between these groups in the other lipid classes.

**Figure 3:**
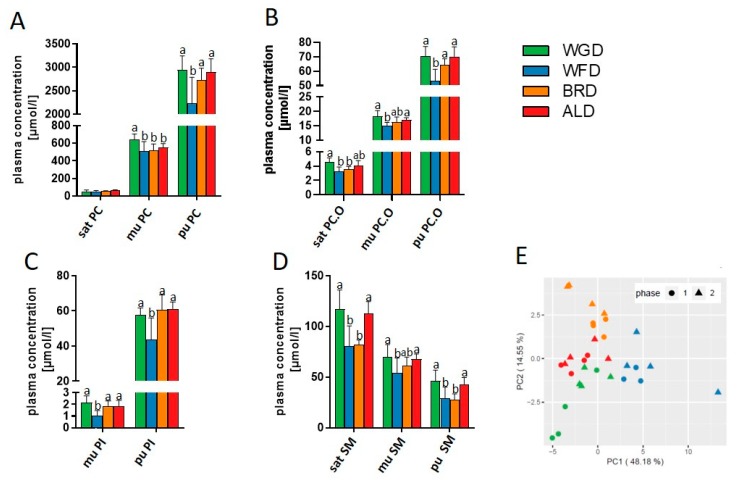
Plasma concentrations of several lipid classes, namely: Phosphatidylcholine (PC) (**A**), PC-ether (PCO) (**B**), phosphatidylinositol (PI) (**C**), and sphingomyelin (SM) (**D**), as well as the PCA results of lipidomics analyses (**E**). Data presented is mean ± SD; *n* = 7–8/group. ALD: Aleurone supplemented diet; BRD: Bran supplemented diet; mu: Monounsaturated; pu: Polyunsaturated; sat: Saturated; WFD; White flour supplemented diet; WGD: Whole grain supplemented diet.

As PC, PCO, PI, and SM were the most affected by the intervention, we analyzed these lipid classes in more detail. Animals receiving the WFD had lower plasma concentrations of monounsaturated and polyunsaturated PI compared to all other groups (Figure 3C). Similarly, the WFD fed mice had lower plasma concentrations of polyunsaturated PC and PCO compared to the other groups (Figure 3A,B). Concentrations of monounsaturated PC were increased in mice of the WGD group compared to all other groups, whereas, for PCO, the differences were only observed between the WGD and WFD. For SM, feeding mice with the WGD and ALD increased plasma concentrations of saturated and polyunsaturated SM compared to the WFD and BRD, but monounsaturated SM were only increased compared to the WFD (Figure 3D). Thus, in contrast to what we observed in the liver, it seems that plasma concentrations of the different phospholipids were similar for the WGD, ALD and BRD, but differed from the WFD.

We then selected the 30 most significant lipids (i.e., those with the lowest *p*-value for the F-test) to perform a principal component analysis (PCA). As depicted in Figure 3E, data points representing mice that received the same intervention diet segregated into non-overlapping groups in the PCA plot, confirming an influence of the type of grain fraction on the composition of plasma lipids.

### 3.3. SCFA Production and Microbiota Composition

**Figure 4:**
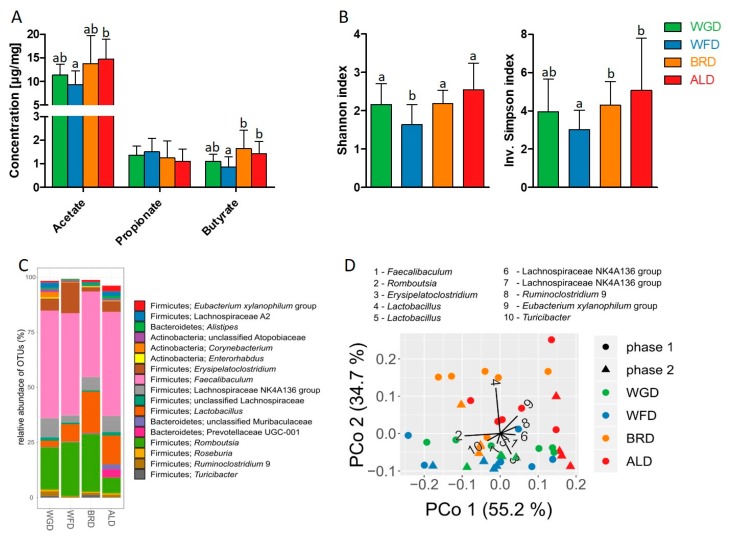
Effect of the different grain fractions on cecal SCFA concentrations (**A**), microbial α-diversity delineated as Shannon and inverse Simpson indices (**B**), average relative abundance of microbial genera (**C**), and microbial β-diversity (**D**) demonstrated using principal coordinates analysis (PCoA) based on Jensen–Shannon-divergence (JSD). Each data point represents an individual mouse with the experimental phase and diet group indicated by the shape and color, as specified in the legend. Black lines indicate the contribution (“loadings”) of each OTU to the principal coordinates. Percentages indicate the fraction of total variance explained by the respective principal coordinate. For bar diagrams: Values shown are means ± SD. ^a, b^ marked bars without a common letter differ significantly (*p* < 0.05).

It has been reported previously [52] that gut microbiota derived SCFA can influence (hepatic) lipid metabolism and are metabolized into longer chain fatty acids. In rodents, the cecum is the organ where most of the microbial fermentation takes place, and so we investigated the concentrations of SCFAs and microbiota composition in the cecum content. Mice that were fed the ALD had increased concentrations of acetate and butyrate in their cecum when compared to the WFD (Figure 4A). Furthermore, feeding of the BRD resulted in increased butyrate concentrations, compared to the WFD. However, no differences in SCFA concentrations were observed between the WGD and the WFD.

The cecal microbiota was dominated by bacteria of the Firmicutes phylum, together with Bacteroidetes and Actinobacteria (Figure 4C). Across all samples, 55 OTUs have been found that exceeded a relative abundance of 1% in at least one of the samples.

Increased α-diversity, the diversity of taxa within a sample, has been associated with better health [53,54,55,56], and thus we assessed the Shannon and inverse Simpson diversity indices. Microbial α-diversity was higher in mice that were fed the WGD, BRD, and ALD, compared to the WFD (Figure 4B). Microbial diversity between communities, (β-diversity) revealed significant differences in the cecal microbiota composition between the different dietary groups (*p* < 0.001, R² = 0.33) in a PERMANOVA test. In a principal coordinates analysis (PCoA) plot (Figure 4D), cecal microbiota separates clearly between animals fed the ALD and BRD on the one hand, and the WFD and WGF on the other. The pairwise comparisons showed differences between all dietary groups (*p* ≤ 0.05), except for the comparison of the WGF and WFD.

In order to identify associations between bacteria and SCFAs, we analyzed the correlations between OTU abundance and SCFA concentrations (Figure S1). Many OTUs were found to be positively correlated with the concentration of butyrate, including *Bacteroides* and three OTUs of *Lactobacillus*. Concentrations of acetate showed a very similar pattern of correlation with OTU abundances (Figure S1). In contrast, the concentration of propionate showed a positive correlation with very few OTUs, including *Faecalibaculum* and *Erysipelatoclostridium*. Notably, the pattern is mostly complementary with the correlation pattern of acetate and butyrate.

In total, the analysis of cecal contents revealed a distinction between the ALD/BRD on the one hand, and the WFD on the other, which can be perceived both in the microbial composition and SCFA concentrations. The WGD in contrast seems to be intermediate, between the three other groups.

## 4. Discussion

Even though wheat is one of the most common grains, especially in Western countries [23], our knowledge on the mechanisms of action related to the beneficial health effects of whole grain wheat and the importance of the different grain fractions therein, is still limited. Thus, the aim of the present study was to investigate the physiological effects of the different wheat fractions in a murine obesity model.

Obesity has been associated with the storage of lipids in the liver and NAFLD, which seems to be a risk factor for the development of type 2 diabetes, even without the manifestation of hepatic consequences [57]. Although the different intervention diets did not impact liver weight, the BRD and ALD reduced triglyceride concentrations in the liver, thus indicating a hepatoprotective effect. Furthermore, the BRD and ALD lead to an increase in expression of SREBF-1 and HSL, compared to the WGD and SCD1, compared to the WGD and WFD. SREBF-1 is an important transcription factor in de novo lipogenesis, and in the uptake of triglycerides in the liver [58]—Which amongst other targets activates the expression of SCD1, the enzyme responsible for the introduction of a double-bond into saturated fatty acids. However, we have not observed an increase in the gene expression of acetyl-coenzyme A carboxylase beta (ACAC-β) and fatty acid synthase (FASN), which are the rate-limiting enzymes in de novo lipogenesis, and also the target genes of SREBF-1 [59]. In addition, the fact that triglyceride concentrations were decreased in the BRD and ALD groups indicates that de novo lipogenesis was not increased, and that, contrarily, hints toward increased lipolysis. This is in line with the observed higher gene expression of HSL, which catalyzes the splitting of triglycerides. Serotonin is known to induce hepatic lipid storage, and the reduction of peripheral serotonin concentrations is discussed to play a role in the prevention of NAFLD [60]. A recent study has shown that consumption of rye bran and wheat aleurone led to reduced serotonin concentrations in plasma and colonic tissue, in humans and mice respectively [61]. Thus, serotonin signaling could be a mechanism that links the consumption of different grain fractions to the regulation of hepatic lipid storage. However, further studies are needed to investigate this aspect in detail.

In summary, our results indicate that even though SREBF-1 expression was higher in mice fed the BRD and ALD, de novo lipogenesis was not affected, and so the BRD and ALD might have only led to an activation of SCD1; which thus, could lead to an increased production of MUFA. Therefore, we next examined the concentrations of long-chain fatty acids in liver phospholipids, but in contrast to our expectations, oleate concentrations were reduced. Further research is needed to investigate the underlying mechanisms.

It has been reported that SCFA, products of microbial fermentation, can impact hepatic liver metabolism [62]. Moreover, it is well known that diet, and especially fiber-rich food, can influence the composition and functionality of the gut microbiota [63]. Thus, we further investigated the impact of our intervention on cecal microbiota composition and concentrations of SCFA. Mice in theWFD group had decreased microbial α-diversity compared to the other three groups. In the last couple of years, it has been shown that the gut microbiota plays a pivotal role in human health [64,65,66].A decreased microbiota α-diversity has been associated with several diseases [53,54,55,56]. Furthermore, it is known that the availability of microbiota-accessible carbohydrates is pivotal for microbial diversity [67,68]. Thus, our results indicate that the lack of dietary fiber in the WFD led to a decrease in microbial diversity, which might have a negative health impact.

A variety of effects have been discussed for SCFAs [13], and especially butyrate has been associated with beneficial health effects, such as the prevention of colorectal cancer [69]. Various mechanisms of actions are discussed for SCFAs. On the one hand they serve as energy substrates, especially for the gut epithelial cells, and on the other hand they are potent signaling molecules, which mediate their effects through G-protein coupled receptors, or through inhibition of histone deacetylases [13]. In an elegant study using labeled SCFAs, it was further shown that acetate and butyrate derived from the cecum are precursors of palmitate synthesis [52]. Moreover, it is being discussed that intestinal microbial load impacts hepatic lipid metabolism by influencing enzyme expression, as a study in germfree mice reported a decreased expression of hepatic SCD1 compared to specific pathogen free mice [62]. However, whether this effect is mediated by SCFAs still needs to be investigated. In light of the current knowledge on gut microbiota and SCFAs and their impact on hepatic liver metabolism, our results indicate that the surplus of dietary fiber in the ALD and BRD groups, in comparison to the WFD group, led to increased microbial diversity and higher SCFA concentrations, which in turn might have increased hepatic expression of SCD1, and altered fatty acid profiles of hepatic phospholipids [60,61]. Further studies are needed to investigate this aspect in more depth.

Furthermore, we observed changes in plasma lipid concentrations. The higher concentrations of unsaturated lipids in plasma could be explained by the alterations in hepatic lipid metabolism. Results for SM are surprising, as the WFD and BRD led to lower concentrations of SM, independent of saturation compared to the WGD and ALD. These results are in contrast to the other observations and warrant further investigation.

Interestingly it has been described previously that aleurone can increase eicosapentaenoic acid plasma concentrations [70], an effect that was associated with aleurone polyphenols. In addition, other studies have shown increased plasma concentrations of PUFA after interventions with polyphenol-rich foods [71,72,73]. Thus, it might be that not only dietary fiber and its impact on intestinal microbiota and SCFAs are involved in mediating wheat effects on lipid metabolism, but that the phytochemicals in wheat might play an important role as well. However further research is needed to investigate the underlying mechanisms.

Even though gut microbiota and lipid metabolism were influenced by the dietary intervention, body weights did not differ significantly between the four intervention groups, despite the fact that the WGD and WFDs contain higher levels of starch, and thus provide more easily accessible energy than the ALD and BRD. However, the ALD and BRD contain more dietary fiber, which can be metabolized to SCFAs, known to be energy substrates as well [13]. Studies comparing the effects of the four wheat fractions used in the present study on body weight and obesity development are lacking, but one study investigated the effect of aleurone on obesity development [74]. In line with our results, body weight was not influenced by aleurone-supplementation of the diet. Further, a systematic review and meta-analysis from 2013 concluded that whole grain consumption does not affect body weight [75]. Thus, our results support the existing literature and indicate that none of the wheat fractions studied improve body weight, despite the higher content of dietary fiber, and thus the lower content of energy in bran and aleurone.

Obesity leads to the storage of surplus energy in the form of triglycerides in various organs, such as adipose tissue [51] and the liver [57]. In adipose tissue this leads to hypertrophy of adipocytes and hypoxia, which can result in necrosis of adipocytes, infiltration of T cells and macrophages, and secretion of proinflammatory cytokines, leading finally to an adipose tissue-associated inflammation [51]. As a low-grade inflammation is involved in the pathogenesis of many obesity-associated diseases [76,77,78], we set out to investigated this aspect. We first of all assessed the impact of the different wheat fractions on adipose tissue weight and adipocyte size, but did not observe any effects. These results are in line with other studies, investigating the effect of aleurone alone, or in comparison with bran on the adipose tissue weight in diet-induced obesity (DIO) mice, which did not detect any effects of these wheat fractions either [74,79]. The effect of whole grain versus refined wheat has been investigated in two human intervention studies. Whereas Schutte et al. [80] reported no effect of either refined or whole grain wheat on the distribution of different adipose tissue depots, nor adipocyte size, Kikuchi et al. [81] observed a decrease in the visceral adipose area in the whole grain compared to the refined grain group. Both studies used the same amount of intervention products, and the same length of intervention periods, but the study from Kikuchi et al. was conducted in Japan and the study from Schutte et al. was conducted in the Netherlands. Thus, variances in background diet and genetics can be assumed, which might explain the different results. As we did not observe any effects on body weight, adipose tissue weight or adipocyte size, we did not further investigate adipose tissue-associated inflammation.

One limitation of this study is that it needed to be conducted in two separate phases. To overcome this limitation, we included the aspect as a fixed effect in our statistical analysis. A further limitation could be that the increase in body weight in our model was too small to actually detect more beneficial effects of the wheat fractions. At the end of the intervention period, the average body weight for all groups was around 36 g, independent of the wheat fraction. In contrast to other studies, in which DIO mice reached average bodyweights of more than 40 g [24,82,83], the mice of the present study remained rather lean. Body weight of aged-matched C57BL/6J mice always fed a low-fat diet has been reported to be approximately 31 g [82], thus we conclude that our diets did induce higher body weight gain than a standard low-fat diet, but final body weights were still on the lower end of obesity. Additional control groups, with a low-fat diet or HFD without a grain fraction supplementation, could provide additional insight for a more conclusive interpretation of the results. However, the primary objective of the study was the comparison of the different wheat fractions, which does not require such additional control groups. One drawback of DIO models is, that the HFDs used to induce obesity vary widely [84]. Whereas, in some studies, the fat content of experimental diets can exceed 70% kcal [85,86], most of the HFDs used contain 40% kcal to 60% kcal [84]. As in humans, dietary fat constitutes approximately 30% of energy intake [87], so we decided to use a HFD from the lower end of the spectrum, with approximately 40% kcal of fat, comparable to diets used before [88]. The use of this moderate, but physiological diet, can explain the relatively low body weights in our model.

In summary, our results could show, that even though the different wheat fractions did not influence body weight in DIO mice, fractions with higher fiber content led to increased gut microbiota diversity and SCFA concentrations, which in turn might have led to the observed effects on lipid metabolism. The decreased concentrations of hepatic triglycerides and the higher hepatic and systemic concentrations of PUFAs indicate a protective effect of bran and aleurone.

## Figures and Tables

**Table 1:** Composition of experimental diets [g/kg].

	WGD	WFD	BRD	ALD
Casein	200	200	200	200
Cornstarch	395	395	395	395
Sucrose	50	50	50	50
Lard	190	190	190	190
Whole grain flour	100			
White flour		100		
Bran			100	
Aleurone				100
Vitamin mix	10	10	10	10
Mineral mix	50	50	50	50
L-Cysteine	3	3	3	3
Choline chloride	3	3	3	3

WGD: Whole grain supplemented diet; WFD: White flour supplemented diet; BRD: Bran supplemented diet; ALD: Aleurone supplemented diet.

**Table 2:** Food intake as well as phenotypic data of C57BL/6J mice fed one of four high-fat diets (HFDs) for 12 weeks.

	WGD	WFD	BRD	ALD
Food intake [g/day]	2.9 ± 0.1	2.9 ± 0.2	3.2 ± 0.4	3.0 ± 0.2
Body weight [g]	35.7 ± 3.3	36.0 ± 3.2	36.8 ± 4.1	36.2 ± 3.2
Liver [g]	1.6 ± 0.2	1.6 ± 0.2	1.5 ± 0.2	1.5 ± 0.2
EAT [g]	1.8 ± 0.3	1.9 ± 0.4	1.9 ± 0.5	1.9 ± 0.5
SAT [g]	1.8 ± 0.6	1.9 ± 0.8	2.4 ± 0.8	2.0 ± 0.5
VAT [g]	0.5 ± 0.2	0.6 ± 0.1	0.6 ± 0.2	0.5 ± 0.2
Adipocyte density [cell/mm^2^]	0.48 ± 0.11	0.51 ± 0.11	0.47 ± 0.10	0.46 ± 0.09

ALD: Aleurone supplemented diet; BRD: Bran supplemented diet; EAT: Epididymal adipose tissue; SAT: Subcutaneous adipose tissue; VAT: Visceral adipose tissue; WFD; White flour supplemented diet; WGD: Whole grain wheat supplemented diet. Data presented is mean ± SD; *n* = 10/group.

**Table 3:** Long-chain fatty acid composition [%] of liver phospholipids from C57BL/6J mice fed the WGD, WFD, BRD or ALD for 12 weeks.

	WGD	WFD	BRD	ALD
C14:0	0.11 ± 0.01	0.11 ± 0.01	0.11 ± 0.02	0.10 ± 0.01
C15:0	0.05 ± 0.01	0.05 ± 0.01	0.06 ± 0.01	0.06 ± 0.01
C16:0	23.05 ± 0.49	23.25 ± 0.49	22.65 ± 0.64	22.54 ± 0.76
C16:1n7c	0.97 ± 0.11	0.98 ± 0.12	1.00 ± 0.16	0.88 ± 0.04
C17:0	0.21 ± 0.02	0.21 ± 0.02	0.23 ± 0.03	0.24 ± 0.02
C18:0	16.68 ± 0.55	16.44 ± 0.55	16.63 ± 0.61	17.04 ± 0.47
C18:1n9c	12.33 ± 0.57 ^a^	12.71 ± 1.42 ^a^	11.47 ± 0.84 ^ab^	10.90 ± 1.01 ^b^
C18:1n7c	2.42 ± 0.27	2.35 ± 0.34	2.42 ± 0.40	2.30 ± 0.38
C18:2n6t	0.09 ± 0.01 ^ab^	0.08 ± 0.01 ^a^	0.09 ± 0.02 ^ab^	0.09 ± 0.01 ^b^
C18:2n6c	7.54 ± 0.70	7.44 ± 0.84	8.24 ± 0.70	7.83 ± 0.94
C18:3n6	0.15 ± 0.03	0.15 ± 0.02	0.15 ± 0.01	0.15 ± 0.02
C18:3n3	0.03 ± 0.01	0.03 ± 0.01	0.03 ± 0.01	0.03 ± 0.00
C20:0	0.19 ± 0.04	0.16 ± 0.04	0.19 ± 0.02	0.20 ± 0.06
C20:1n9	0.31 ± 0.02	0.30 ± 0.04	0.30 ± 0.03	0.29 ± 0.03
C20:2n6	0.23 ± 0.02	0.21 ± 0.03	0.24 ± 0.02	0.23 ± 0.02
C20:3n6	2.47 ± 0.18	2.24 ± 0.23	2.42 ± 0.23	2.29 ± 0.21
C20:4n6	21.61 ± 1.14	21.42 ± 0.85	21.58 ± 0.82	22.22 ± 0.43
C20:5n3	0.11 ± 0.02	0.11 ± 0.03	0.13 ± 0.02	0.11 ± 0.02
C22:4n6	0.38 ± 0.02 ^a^	0.39 ± 0.03 ^a^	0.36 ± 0.02 ^b^	0.38 ± 0.01 ^ab^
C22:5n6	1.67 ± 0.10 ^a^	1.57 ± 0.14 ^ab^	1.36 ± 0.10 ^c^	1.42 ± 0.15 ^bc^
C22:5n3	0.45 ± 0.01	0.46 ± 0.04	0.45 ± 0.02	0.47 ± 0.02
C22:6n3	8.96 ± 0.72 ^a^	9.34 ± 0.37 ^a^	9.87 ± 0.61 ^b^	10.25 ± 0.51 ^b^
∑ n3 FA	9.54 ± 0.71 ^a^	9.95 ± 0.34 ^a^	10.49 ± 0.62 ^b^	10.86 ± 0.51 ^b^
∑ n6 FA	34.14 ± 0.75	33.51 ± 1.13	34.45 ± 1.01	34.61 ± 1.08
n6/n3 FA	3.59 ± 0.27 ^a^	3.37 ± 0.15 ^ab^	3.29 ± 0.17 ^b^	3.19 ± 0.22 ^b^

^a, b, c^ marked values without a common letter in a row indicates significant differences (*p* < 0,05). Data presented is mean ± SD; *n* = 7–8/group. ALD: Aleurone supplemented diet; BRD: Bran supplemented diet; WFD; White flour supplemented diet, WGD: Whole grain supplemented diet.

## Supplementary Materials

The following are available online at https://www.mdpi.com/2072-6643/11/10/2348/s1, **Figure S1:** Correlation of cecal, SCFA concentrations and relative abundance of OTUs. **Table S1:** Oligonucleotides used in this study to measure mRNA levels, **Table S2:** Primer used for microbiota analysis.

## Abbreviations

ACAC-β	Acetyl-coenzyme A carboxylase beta
ALD	Aleurone supplemented diet
BRD	Bran supplemented diet
CPT1α	Carnitine palmitoyltransferase 1 alpha
DIO	Diet-induced obesity
ELOVL5	Fatty acid elongase 5
FASN	Fatty acid synthase
FID	flame ionization detector
HFD	high fat diet
HSL	Hormone-sensitive lipase
MUFA	Monounsaturated fatty acids
NAFLD	Non-alcoholic fatty liver disease
PUFA	Polyunsaturated fatty acids
SCFA	Short-chain fatty acids
SCD1	Stearoyl-CoA desaturase 1
SFA	Saturated fatty acids
SREBF-1	Sterol regulatory element-binding transcription factor 1
WFD	White wheat flour supplemented diet
WGD	Whole grain wheat supplemented diet
