# Supplementary material for: Specific Wheat Fractions Influence Hepatic Fat Metabolism in Diet-Induced Obese Mice
Source: Nutrients. 2019 Oct 2;11(10):2348. doi: 10.3390/nu11102348 (PMC6836242; doi:10.3390/nu11102348)
Supplement: Supplementary file 1 [file nutrients-11-02348-s001.pdf]

Supplementary Materials

Table S1: Oligonucleotides used in this study to measure mRNA levels.

Primer name		Primer sequence (5′→3′)
18S rRNA	f	CTT AGA GGG ACA AGT GGC GTT C
	r	CGC TGA GCC AGT CAG TGT AG
18S rRNA	f	ACC ACA TCC AAG GAA GGC AG
	r	TTT TCG TCA CTA CCT CCC C
	p	6-FAM-AGG CGC GCA AAT TAC CCA CTC CC-TAMRA
Srebf1	f	GAG GAT AGC CAG GTC AAA GC
	r	AGG ATT GCA GGT CAG ACA CA
	p	6-FAM- CCA GCA TGC CTC GGC TGT GT-TAMRA
Pparγ	f	GCT CAA GTA TGG TGT CCA TGA GAT C
	r	TGA GAT GAG GAC TCC ATC TTT ATT CA
	p	6-FAM-ACA CGA TGC TGG CCT CCC TG-TAMRA
Acacβ	f	GCC TCT TCA TCA CCA ACG AG
	r	AAA GAG AGC CTG CCT GAA CA
	p	6-FAM-AGG CAC AGT CCC TCG GGA CC-TAMRA
Fasn	f	TTG ATG ATT CAG GGA GTG GA
	r	TTA CAC CTT GCT CCT TGC TG
	p	6-FAM-CAT AGA CCC GCC GAG CCA GG-TAMRA
Scd1	f	TTC TTC TCT CAC GTG GGT TG
	r	CGG GCT TGT AGT ACC TCC TC
	p	6-FAM-CGC AAA CAC CCG GCT GTC AA-TAMRA
Elovl5	f	GGT GGC TGT TCT TCC AGA TT
	r	CCC TTC AGG TGG TCT TTC C
Cpt1α	f	CCA AAC CCA CCA GGC TAC A
	r	GCA CTG CTT AGG GAT GTC TCT ATG
Hsl	f	TGC TTG GTT CAA CTG GAG AG
	r	GTA ACT GGG TAG GCT GCC AT
	p	6-FAM-CTG CTG CCC GAA GGG ACA CA-TAMRA

Table S2: Primer used for microbiota analysis

1) Enrichment PCR			
Purpose	Name	Full Primer sequence	Reference
Fwd primer	27F	AGAGTTTGATCMTGGCTCAG	Lane (1991)
Rev primer	338R		Etchebehere & Tiedje (2005)
2) Custom barcode integration PCR			
Purpose	Name	Full Primer sequence (barcode sequence in bold)	Reference
Fwd primer + Barcode	IlluFBC1	ACACTCTTTCCCTACACGACGCTCTTCCGATCT**AAGCCT**CAAGAGTTTGATCMTGGCTCAG	Camarinha-Silva et al. (2014)
	IlluFBC6	ACACTCTTTCCCTACACGACGCTCTTCCGATCT**ACCAAT**CAAGAGTTTGATCMTGGCTCAG	Camarinha-Silva et al. (2014)
	IlluFBC12	ACACTCTTTCCCTACACGACGCTCTTCCGATCT**AGAAGC**CAAGAGTTTGATCMTGGCTCAG	Camarinha-Silva et al. (2014)
	IlluFBC19	ACACTCTTTCCCTACACGACGCTCTTCCGATCT**ATAGAC**CAAGAGTTTGATCMTGGCTCAG	Camarinha-Silva et al. (2014)
	IlluFBC21	ACACTCTTTCCCTACACGACGCTCTTCCGATCT**CAGATG**CAAGAGTTTGATCMTGGCTCAG	Camarinha-Silva et al. (2014)
	IlluFBC24	ACACTCTTTCCCTACACGACGCTCTTCCGATCT**CCAGGT**CAAGAGTTTGATCMTGGCTCAG	Camarinha-Silva et al. (2014)
	IlluFBC28	ACACTCTTTCCCTACACGACGCTCTTCCGATCT**CGAATT**CAAGAGTTTGATCMTGGCTCAG	Camarinha-Silva et al. (2014)
	IlluFBC33	ACACTCTTTCCCTACACGACGCTCTTCCGATCT**CTAAGA**CAAGAGTTTGATCMTGGCTCAG	Camarinha-Silva et al. (2014)
	IlluFBC37	ACACTCTTTCCCTACACGACGCTCTTCCGATCT**GACCTT**CAAGAGTTTGATCMTGGCTCAG	Camarinha-Silva et al. (2014)
	IlluFBC40	ACACTCTTTCCCTACACGACGCTCTTCCGATCT**GCATCC**CAAGAGTTTGATCMTGGCTCAG	Camarinha-Silva et al. (2014)
	IlluFBC45	ACACTCTTTCCCTACACGACGCTCTTCCGATCT**GGCTTC**CAAGAGTTTGATCMTGGCTCAG	Camarinha-Silva et al. (2014)
	IlluFBC47	ACACTCTTTCCCTACACGACGCTCTTCCGATCT**GTAATC**CAAGAGTTTGATCMTGGCTCAG	Camarinha-Silva et al. (2014)
	IlluFBC51	ACACTCTTTCCCTACACGACGCTCTTCCGATCT**TACGGT**CAAGAGTTTGATCMTGGCTCAG	Camarinha-Silva et al. (2014)
	IlluFBC54	ACACTCTTTCCCTACACGACGCTCTTCCGATCT**TCATAA**CAAGAGTTTGATCMTGGCTCAG	Camarinha-Silva et al. (2014)
	IlluFBC59	ACACTCTTTCCCTACACGACGCTCTTCCGATCT**TGGTCC**CAAGAGTTTGATCMTGGCTCAG	Camarinha-Silva et al. (2014)
	IlluFBC60	ACACTCTTTCCCTACACGACGCTCTTCCGATCT**TTATGC**CAAGAGTTTGATCMTGGCTCAG	Camarinha-Silva et al. (2014)
Rev primer	IlluRevAdap	GTGACTGGAGTTCAGACGTGTGCTCTTCCGATCTTGCTGCCTCCCGTAGGAGT	Camarinha-Silva et al. (2014)

3) Illumina multiplexing PCR			
Purpose	Name	Full Primer sequence (index sequence in bold)	Reference
Fwd primer	Multiplexing_ PCR	AATGATACGGCGACCACCGAGATCT	Camarinha-Silva et al. (2014)
Rev primer + index	Index_1	CAAGCAGAAGACGGCATACGAGAT**CGTGAT**GTGACTGGAGTTC	Illumina
	Index_2	CAAGCAGAAGACGGCATACGAGAT**ACATCG**GTGACTGGAGTTC	Illumina
	Index_3	CAAGCAGAAGACGGCATACGAGAT**GCCTAA**GTGACTGGAGTTC	Illumina
	Index_4	CAAGCAGAAGACGGCATACGAGAT**TGGTCA**GTGACTGGAGTTC	Illumina
	Index_5	CAAGCAGAAGACGGCATACGAGAT**CACTGT**GTGACTGGAGTTC	Illumina
	Index_6	CAAGCAGAAGACGGCATACGAGAT**ATTGGC**GTGACTGGAGTTC	Illumina
	Index_7	CAAGCAGAAGACGGCATACGAGAT**GATCTG**GTGACTGGAGTTC	Illumina
	Index_8	CAAGCAGAAGACGGCATACGAGAT**TCAAGT**GTGACTGGAGTTC	Illumina
	Index_9	CAAGCAGAAGACGGCATACGAGAT**CTGATC**GTGACTGGAGTTC	Illumina
	Index_10	CAAGCAGAAGACGGCATACGAGAT**AAGCTA**GTGACTGGAGTTC	Illumina
	Index_11	CAAGCAGAAGACGGCATACGAGAT**GTAGCC**GTGACTGGAGTTC	Illumina
	Index_12	CAAGCAGAAGACGGCATACGAGAT**TACAAG**GTGACTGGAGTTC	Illumina

Figure S1: Correlation of cecal SCFA concentrations and relative abundance of OTUs.

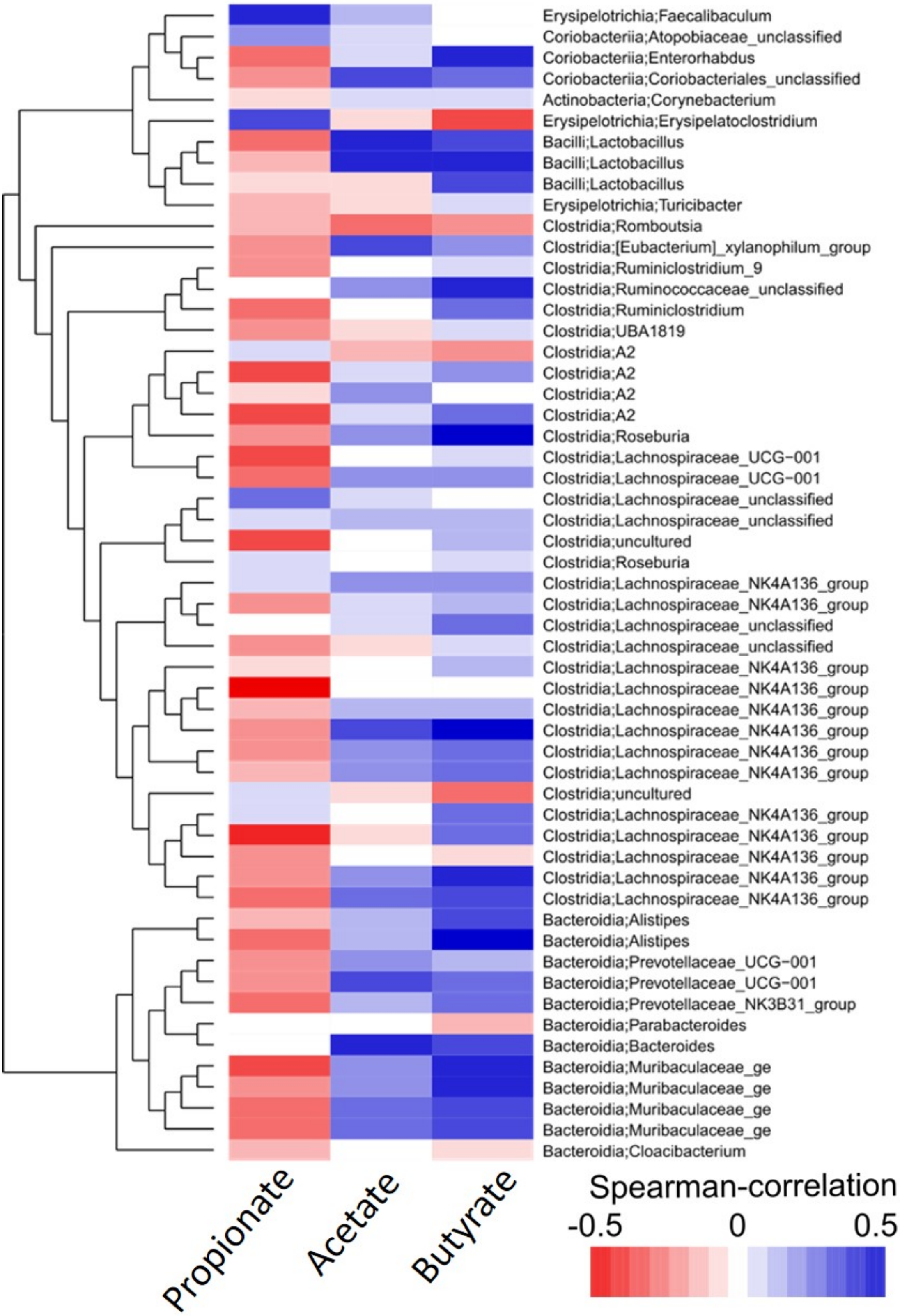

The heatmap of spearman correlation coefficients was ordered vertically according to a phylogenetic tree of the OTUs consensus sequences to approximately reflect evolutionary relatedness.
